# Allopathic versus Homeopathic Strategies and the Recurrence of Prescriptions: Results from a Pharmacoeconomic Study in Italy

**DOI:** 10.1093/ecam/nep023

**Published:** 2010-10-21

**Authors:** Andrea Basili, Francesco Lagona, Paolo Roberti di Sarsina, Corallina Basili, Teresa Valeria Paterna

**Affiliations:** ^1^Italian Society of Anthroposophic Medicine, Department of Public Institutions, Economy and Society, University of Roma Tre and Max Planck Institute for Demographic Research, Rome, Italy; ^2^Department of Public Institutions, Economy and Society, University of Roma Tre, Via G. Chiabrera 199 - 00146 Rome, Italy; ^3^Expert for CAM, Italian High Council of Health, Ministry of Health, Rome, Italy

## Abstract

This is a pharmaeconomic study to assess the impact of different, cost-specific pharmacological strategies on the recurrence rate of prescriptions in the treatment of cold symptoms. Data were obtained from a prospective cohort study reporting individual prescriptions histories of subjects experiencing cold symptoms, obtained by a stratified random sample of 316 subjects, clustered into 139 Italian families, followed up for 40 months. Costs of homeopathic and allopathic treatments were recorded within each prescription. A Cox proportional hazards model with random effects was exploited to regress time elapsed between subsequent prescriptions over the relative difference between homeopathic- and allopathic-related costs, adjusting for age and gender and accounting for unobserved individual heterogeneity. Relative risks of event (prescription) re-occurrence have been estimated. The recurrence rate of prescriptions raise when allopathic strategies are preferred to homeopathic alternatives. No significant differences were observed between gender groups, while age was marginally significant. Inter-subjects heterogeneity was not significant.

## 1. Introduction

Studies on cost-effectiveness of homeopathy aim to show that homeopathic cure strategies are less expensive than conventional medicine and that health costs could be lowered if mainstream therapies would be replaced by homeopathy. For example [1], a decrease in admissions to hospital outpatient services and pharmaceutical utilization has been reported when complementary medicine was used in conjunction with conventional medicine. However, some data have suggested that including complementary medicine may increase overall costs for adults as it is used as an “add on” rather than a replacement [2].

These studies are often carried out by comparing medical effectiveness, quality of life and costs resulting from patients who undergo a homeopathic treatment, to the outcomes from a control group, typically treated by conventional mainstream medicine. Although cost data should be collected prospectively, as a part of a pragmatic clinical trial [3–6], these studies are time-consuming and quite expensive. As a result, the literature on the cost-effectiveness of homeopathy is often based either on *post-hoc* analysis of past prospective studies [7] or on retrospective studies [8] or comparing the costs of homeopathic treatments in a group to the (estimated) cost of conventional drugs which otherwise would be prescribed for the observed subjects [9].

Fewer are prospective cohort studies that compare cost-effectiveness of allopathic and homeopathic treatments. The outcomes research by Witt etal. [10] for example, is based on a prospective, non-randomized, open cohort study, where subjects are approached at the doctor's practice and thus already made their own choice of therapy (purely allopathic or purely homeopathic). The authors' conclusion was that patients seeking homeopathic treatment had a better outcome (in terms of severity of symptoms) overall compared with patients on conventional treatment, whereas total costs in both groups were similar.

In everyday medical practice, though, patients' choices are seldom consistent with a pure pharmacological strategy. Typically, patients exploits homeopathy as an add-on treatment, in conjunction with conventional medicine. Moreover, some subjects who started a pure allopathic therapy may change their mind and turn to homeopathic strategies, while others unsatisfied with homeopathy may decide to go back to allopathic strategies. In the case of such time-varying, mixed strategies, it is difficult to ascertain if patients' benefits are associated with a kind of therapy and its cost. Observational studies that account for heterogeneity in patients' behavior are, nevertheless, of special interest to insurers and medical service providers. However, to our knowledge, no study has provided results on both the outcome and costs of treatment, when subjects are left free to change their pharmacological strategy, during the follow up.

In this article, the outcome of interest is the temporal interval between any two subsequent prescriptions involving either allopathic or homeopathic or mixing allopathic and homeopathic treatments, during a follow up of subjects who experience cold symptoms. The rate at which prescriptions occur within the subject's history is important to evaluate the effectiveness of a treatment. For example, long intervals between consecutive prescriptions may indicate that, while the subject's pharmacological strategy stops suddenly the symptoms, it does not avoid the re-occurrence of the same symptoms after a long period of time. Conversely, short intervals followed by long intervals may indicate, on one side, that the treatment involves a number of prescriptions to have an appreciable impact on the subject, and that, on the other side, the strategy of choice reduces the risk of re-occurrences of symptoms.

For each inter-prescription interval, we have considered the cumulative cost of both homeopathic and allopathic treatments charged to each subject up to the date of the latest prescription. The relative difference between the (cumulative) costs attributable to the allopathic treatments and those relating to the homeopathic strategy was computed to summarize the subject-specific, time-varying economic relevance of one strategy over the other one, or, in other words, an indicator of the financial weight given by a subject to each strategy during the follow up.

Regressing the observed inter-prescription intervals on this indicator can help testing the impact that the financial effort for a specific pharmacological history has on the rate of prescriptions occurrences. We modeled the rate at which prescriptions occurred by exploiting a Cox proportional hazards model [11]. Under the simplifying assumption that the rate at which precriptions occurr varies proportionally with the economic relevance of a pharmacological strategy, this approach allows to estimate the effect of a treatment's relative cost on the rate of medical interventions. We also extended this analysis by including a random effect [12] and thereby adjusting for the presence of possibly unobserved confounding factors.

Our pharmacoeconomic study has been carried out by prospectively collecting the biographies (dates and costs of prescriptions) of 316 subjects belonging to 139 Italian families, drawn from the list of Italian journalists' families by a stratified random sampling design and followed up for 40 months. Among these, only 82 biographies reported two or more cold-related prescriptions and were included in the analysis.

## 2. Materials and Methods

### 2.1. Data

CASAGIT is the journalists' health insurance company in Italy. This company allows for reimbursement of both allopathic drugs and homeophatic prescriptions bought by its customers and their families (the study population). A stratified samplingschemewas adopted to draw a sample of 139 families (316 patients) for follow up. Sample size was determined according to the available funds for the present project. Stratification variables included the average age of the family, residence and family size. All the subjects within each family were followed up during the period June 2002 to October 2005, after giving written informed consent. The study was compliant with relevant data protection laws.

For each subject, the dates of the prescriptions were recorded. The treatments prescribed each time were clustered into two classes, namely conventional or homeopathic, and the relating costs recorded. Homeopathic treatments included purely homeopathic, antroposophical and homeo-tossicological remedies. In keeping with other studies [10], total costs of homeopathic and allopathic treatments were similar.

**Figure 1:**
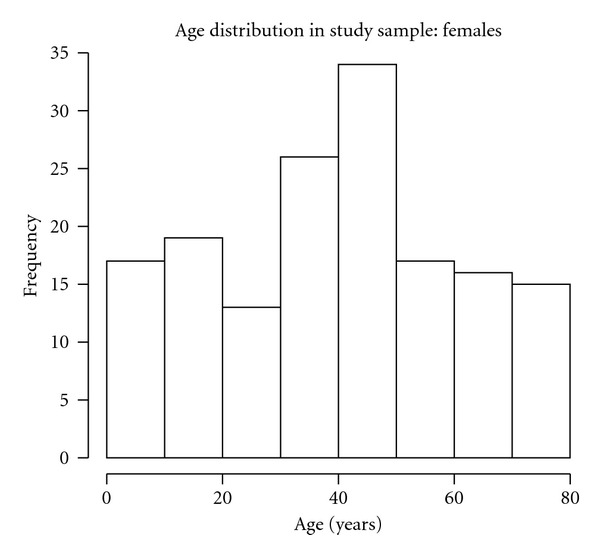
Age distribution of the 174 females in the study sample.

**Figure 2:**
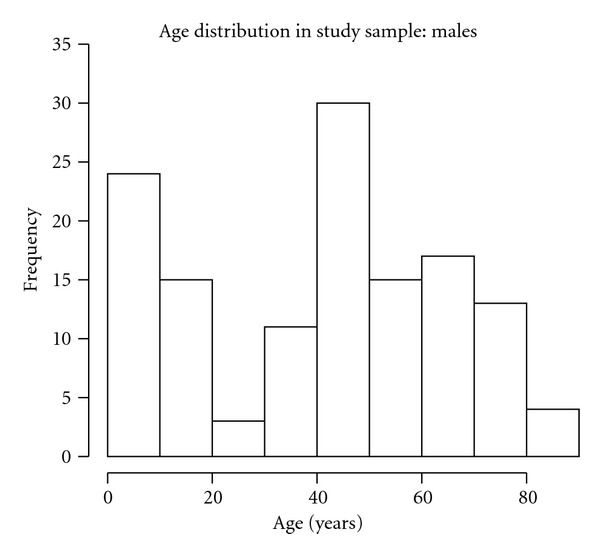
Age distribution of the 141 males in the study sample.

Gender and age were also included in the individual profile. About 55% of the sampled subjects were females. Figures 1 and 2 display the age distribution of the sample. We notice that the age classes between 20 and 30 years contain a few subjects, as expected when a journalist's family is chosen as sampling unit. Only 151 subjects (among the 316 included in the sample) experienced cold-related symptoms (common cold, influenza and para-influenza), without co-morbidities such as chronic pulmonary diseases or chronic cardiovascular diseases, as reported by the family GP. Among these, only the 82 subjects who, during the follow up, asked for reimbursement of two or more prescriptions were considered for further statistical analysis, as we are interested in re-occurrence of prescriptions.

**Figure 3:**
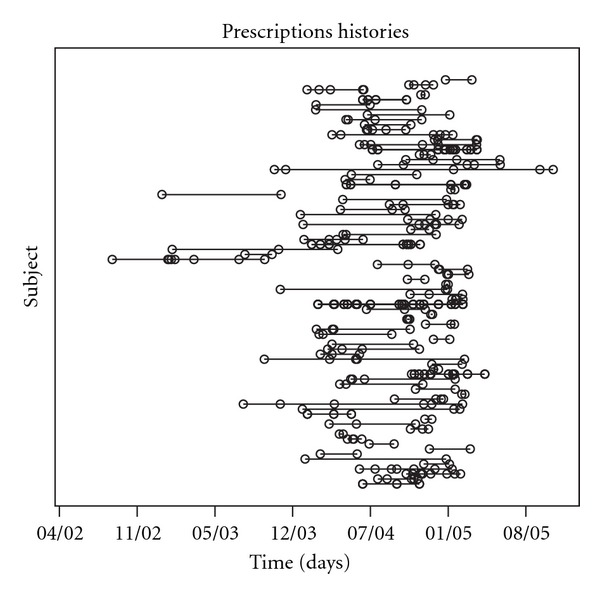
Prescription histories of the 82 subjects experiencing cold symptoms in the study sample, during the follow up (period June 2002 to October 2005).

Figure 3 depicts the intervals between any two subsequent prescriptions within each of the 82 subjects, during the follow up. The dots in the picture represent the 231 observed prescriptions: of these, 67 (29%) were purely allopathic, 27 (12%) were purely homeopathic, while the remaining prescriptions involved a mixture of homeopathic and allopathic pharmacological treatments.

The large variability of the lengths of these intervals is apparent, showing different typical patterns of events. Specifically, some subjects experience a number of prescriptions occurring in a short period of time, hence separated by short intervals, and no prescriptions afterwards. Others exhibit single-event occurrences separated by longer intervals. Finally, a part of the sample shows mixed patterns, featuring short inter-prescription intervals, followed by longer intervals and, conversely, distant event occurrences followed by short intervals.

### 2.2. Statistical Analysis

The main goal of the statistical analysis exploited in this study was to test the impact of the economic relevance of allopathic versus homeopathic strategies on the prescriptions patterns experienced by the observed subjects.

For each subject, the number of days between any two prescriptions was calculated. We also evaluated the cumulative costs of allopathic and homeopathic prescriptions occurred up to the right end of each interval. The relative difference between allopathic and homeopathic cumulative costs was calculated for each interval: this cost-incidence index takes the value of −1 for a subject following a pure homeopathic therapy up to the date relating to the right end of the interval and +1, in case of a pure allopathic therapy. When the costs of the two strategies are equal, the index takes the value 0. Negative (positive) values indicate that the cumulative homeopathic costs were larger (lower) than their allopathic counterpart. This index is a continuous measure of cumulative costs incidence that avoids a naive repartition of subjects into three groups with a debatable homogeneity: (i) receiving only allopathy, (ii) receiving only homeopathy and (iii) receiving both.

A Cox proportional hazards model [11] was fitted to the durations between prescriptions by using the cumulative relative difference between allopathic and homeopathic cumulative costs as the main covariate, and simultaneously adjusting for age and gender. After testing the proportional hazards assumption [12] relative risks were estimated with respect to subjects whose cost-incidence index was equal to 0 (baseline group).

A key issue when analyzing biographies of a number of subjects is inter-subjects heterogeneity due to unobserved confounding factors. In our case study, there could be a number of unobserved factors (such us a patient's compliance with precriptions and practictioner's habits) that could bias the outcome of a statistical analysis that does not account for possible heterogeneity. One way to investigate and accommodate this heterogeneity is to use models that include random effects. We thereby generalized our analysis by fitting a Cox model with a random intercept [12]. This approach allows (i) to adjust risk estimates by unobserved heterogeneity and, simultaneously, (ii) to test the significance of the amount of heterogeneity found in the data, through a Chi-squared test on the variance of the random effect.

## 3. Results

### 3.1. Relative Incidence of Costs on the Recurrence of Precriptions

**Figure 4:**
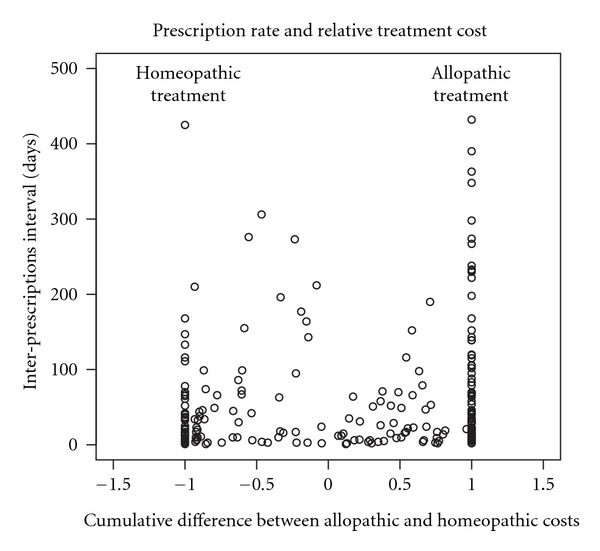
Inter-prescription intervals versus the relative cumulative difference between allopathic and homeopathic costs.

Figure 4 shows the relationship between the relative incidence of allopathic costs on homeopathic costs and durations between prescriptions.

**Figure 5:**
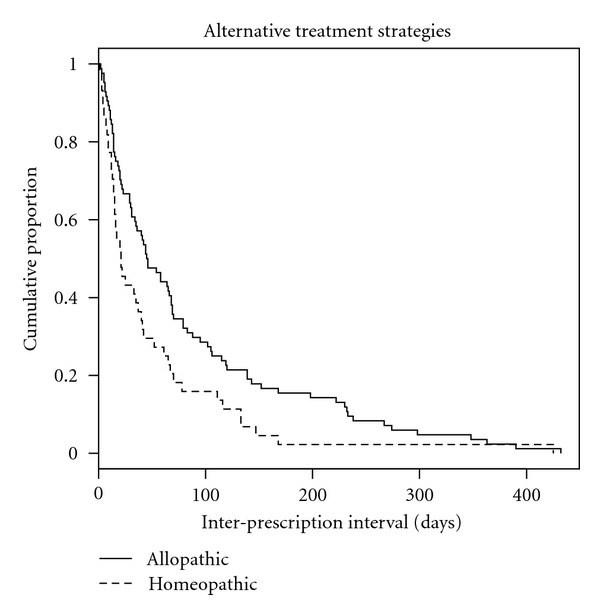
Cumulative proportions of inter-prescriptions intervals exhibited by subjects undergoing a pure allopathic strategy (solid line) and a pure homeopathic strategy (dashed line).

Intervals featuring a pure homeopathic treatment are on average shorter than intervals characterized by allopathic prescriptions. Interestingly, the variability of durations seems to increase only when allopathic costs are larger then their homeopathic counterpart, reaching a maximum when a pure allopathic strategy is undergone. For illustratory purposes, a comparison between the intervals distribution under a pure homeopathic and a pure allopathic strategy is shown by Figure 5: the risk of large intervals between prescriptions is lower for subjects undergoing homeopathic treatments than that experienced by subjects under a conventional allopathic treatment.

### 3.2. Risks of Precriptions Re-Occurrences

Figure 5 shows some evidence of the impact that cost incidence seems to have on the length of the intervals between any two subsequent prescriptions, but it does not allow a clear-cut conclusion. We hence fitted a Cox proportional hazards model where the re-occurrences rate is regressed on the relative difference between allopathic and homeopathic cumulative costs, simultaneously adjusting for the available confounding factors (age and gender). Tests [13] of hazards proportionality were statistically significant on each covariate—*P*-values:  .962 (age),  .382 (gender) and  .350 (relative cost incidence).  Table 1 reports the resulting estimates of risks with 95% confidence intervals.

Remarkably, no significant differences were found between gender groups. The marginally significant effect of age indicates that the risk of re-occurrence of prescriptions later in time may increases up to 1.2% for each year of life, no matter which treatment strategy is chosen. After adjusting for age and gender, the impact of relative allopathic cost incidence is still significant and the estimate of Table 1 shows that decreasing the relative (economic) incidence of allopathic drugs by 1%, reduces the risk of prescription re-occurrences by 18% (±12% at 95% confidence level).

### 3.3. Accounting for Unobserved Confounding Factors

Although encouraging, the outcomes reported in Table 1 could be biased in the case of important confounding factors that were not observed during the present study. In other words, it is possible that subjects are heterogeneus with respect to one or more unobserved covariates that could have a significant influence on the response variable.  Table 2 displays the estimates obtained after including a random effect in the Cox proportional hazards model, to account for latent heterogeneity.

The estimated variance of the random effect was equal to  .093 (*P* =  .110), suggesting that the subjects in the study sample were not significantly heterogeneous. The outcomes obtained without the inclusion of a random effect (Table 1) are essentially confirmed after accounting for latent heterogeneity. In particular, the influence of relative allopathic cost incidence is still significant, although with a wider confidence interval than that obtained without including a random effect.

## 4. Discussion

Studies on cost effectiveness of homeopathy are usually based on comparing outcomes such as current severity of symptoms and quality of life indexes between a group of patients who undergo homeopathic treatment and a group of subjects treated by conventional medicine. The alternative approach pursued in this article was based on taking the rate at which prescriptions occur in a subject as the outcome of interest, leaving the sampled subjects free to change treatment strategy during the follow up. We found that the rate of event occurrences (prescriptions) can be predicted by the cumulative history of prescription costs experienced by a subject. Specifically, patients who favor homeopathy experience a reduced risk of prescriptions re-occurrence, compared to those who favor an allopathic pharmacological strategy.

Caution is however necessary in the interpretation of this result. First, our results cannot be generalized to the Italian population, since we considered a cohort of journalists' families and the age distribution of the sample does not emulate that of the Italian population. Secondly, homeopathy and conventional medicine were in this article compared according to their costs and, as a result, we assume that a strategy is favored over the other when a strategy's cost is higher than the cost of the alternative strategy. Third, this research was based on cold symptoms, to collect a reasonable number of cases in a follow up of 40 months, and cannot be generalized to other types of symptoms. Fourth, our study did not include the observation of possible confounding factors such as subjects' compliance with prescriptions, practictioners habits, average duration of therapies and daily therapy costs. We tested our estimates for the presence of significant heterogeneity and we found that our sample is not significantly heterogeneous. However, it is possible that the homogeneity found in the data is the result of a follow up of limited length (40 months).

Under the assumption that an observed prescription interval is a marker for cold symptom recurrence, our analysis would offer evidence that a homeopathic strategy is associated with a reduced risk of symptoms recurrence. Although this hypothesis is likely in the present case study, we should not ignore that our analysis is based on prescriptions whose costs were reimbursed by the insurance company, hence excluding possible events which were not reported to the company. Furthermore, our study lacks of data on medical effectiveness, such as benefits experienced by the patients, either self-reported or reported by physicians [10, 12]. For these reasons, the hypothesis that nonoccurrence of new prescriptions in a subject's history can be interpreted as nonoccurrence of further symptoms remains an assumption and cannot be rigorously tested on the basis of the available data.

Although with these limitations, our analysis shows evidence that, while total costs of homeopathic and allopathic pharmacological strategies are similar in a history of cold symptoms, the rate at which prescriptions occurr raise when allopathic strategies are preferred to homeopathic alternatives.

## Funding

IMO, WALA, WELEDA, BOIRON, HEEL and the Department of Public Institutions, Economy and Society of the University of Roma Tre.

## Figures and Tables

**Table 1:** Cox regression of inter-prescriptions durations.

Covariate	Exp(coef)	Lower 0.95	Upper 0.95
Age at prescription (years)	1.006	1.000	1.012
Male (ref: female)	1.161	0.893	1.511
Allopathic relative incidence	0.817	0.699	0.955

**Table 2:** Random effect Cox regression of inter-prescriptions durations.

Covariate	Exp(coef)	Lower 0.95	Upper 0.95
Age at prescription (years)	1.006	1.000	1.013
Male (ref: female)	1.192	0.874	1.627
Allopathic relative incidence	0.820	0.681	0.987
